# Esketamine in depression: putative biomarkers from clinical research

**DOI:** 10.1007/s00406-024-01865-1

**Published:** 2024-07-13

**Authors:** Jenessa N. Johnston, Carlos A. Zarate, Mark D. Kvarta

**Affiliations:** Experimental Therapeutics and Pathophysiology Branch, National Institute of Mental Health, National Institutes of Health, 10 Center Drive, Bethesda, MD 20892 USA

**Keywords:** Ketamine, Biomarkers, Mechanism of action, Neuroimaging, Treatment-resistant depression, Suicide

## Abstract

The discovery of racemic (*R*,* S*)-ketamine as a rapid-acting antidepressant and the subsequent FDA approval of its (*S*)-enantiomer, esketamine, for treatment-resistant depression (TRD) are significant advances in the development of novel neuropsychiatric therapeutics. Esketamine is now recognized as a powerful tool for addressing persistent symptoms of TRD compared to traditional oral antidepressants. However, research on biomarkers associated with antidepressant response to esketamine has remained sparse and, to date, has been largely extrapolated from racemic ketamine studies. Genetic, proteomic, and metabolomic profiles suggest that inflammation and mitochondrial function may play a role in esketamine’s antidepressant effects, though these preliminary results require verification. In addition, neuroimaging research has consistently implicated the prefrontal cortex, striatum, and anterior cingulate cortex in esketamine’s effects. Esketamine also shows promise in perioperative settings for reducing depression and anxiety, and these effects appear to correlate with increased peripheral biomarkers such as brain-derived neurotrophic factor and serotonin. Further indications are likely to be identified with the continued repurposing of racemic ketamine, providing further opportunity for biomarker study and mechanistic understanding of therapeutic effects. Novel methodologies and well-designed biomarker-focused clinical research trials are needed to more clearly elucidate esketamine’s therapeutic actions as well as biologically identify those most likely to benefit from this agent, allowing for the improved personalization of antidepressant treatment.

## Introduction

The discovery of racemic (*R*,* S*)-ketamine (hereafter referred to as ‘ketamine’) as a rapid-acting antidepressant—and the subsequent regulatory approvals for treatment-resistant depression (TRD) and suicidal ideation of its (*S*)-enantiomer, esketamine, by the US Food and Drug Administration (FDA) and other major pharmaceutical regulatory agencies around the world—ushered in a new era for psychiatric therapeutic development. Ketamine, canonically an N-methyl-D-aspartate receptor (NMDAR) antagonist, was first synthesized by Parke-Davis in 1956 and developed in 1962 as a short-acting sedative to replace phencyclidine. In the ensuing decades of the 20th century, it was primarily used as an anesthetic in adult, pediatric, and veterinary settings [1]. Given the potential anesthetic and dissociative effects associated with ketamine use and abuse, its distribution is controlled in Schedule III of the Controlled Substances Act in the United States, though the World Health Organization (WHO) does not currently recommend its classification as a scheduled substance.

A surge of preclinical research into the potential antidepressant effects of MK-801, another NMDAR antagonist [2], spurred reevaluation of ketamine’s potential antidepressant effects. In a seminal clinical study, Berman and colleagues demonstrated that an intravenous (IV) ketamine infusion alleviated symptoms of depression within hours [3], a finding that was subsequently confirmed in TRD and bipolar depression [4, 5]. Since these initial findings, multiple large-scale randomized controlled trials (RCTs) have confirmed ketamine’s effectiveness as a rapid-acting antidepressant with both a clinical response and apparent neurobiological pathway distinct from traditional antidepressant pharmacology [6]. In 2019, FDA approval of Spravato, an intranasal (IN) formulation of ketamine’s (*S*)-enantiomer, broadened ketamine’s use as an antidepressant throughout the medical community; its relative success, despite implementation barriers, has inspired further pharmaceutical development beyond the oral “me-too” medications that have long dominated this space.

Ketamine is highly water- and lipid-soluble, allowing it to rapidly cross the blood-brain barrier [7]. Its binding site is located deep within the NMDAR channel, necessitating activation of the receptor and removal of the magnesium block before ketamine can exert its antagonism [8] through an open-channel block that prevents the movement of ions through the channel [9]. Ketamine is metabolized within minutes through nitrogen-mediated demethylation driven primarily by the action of cytochrome P450 liver enzymes [10, 11]. Norketamine, ketamine’s first major metabolite, can subsequently be metabolized to either hydroxynorketamine (HNK) or dehydroxynorketamine (DHNK). With a half-life of around two to three hours, the elimination of ketamine is around equal to that of liver blood flow (12-20 ml/min/kg) [12, 13], though some research suggests that women have an approximately 20% higher clearance rate [14]. Ketamine’s antidepressant effects last significantly longer than its NMDAR blockade and rapid clearance, suggesting the activation of downstream signaling cascades that cause lasting effects.

**Fig. 1:**
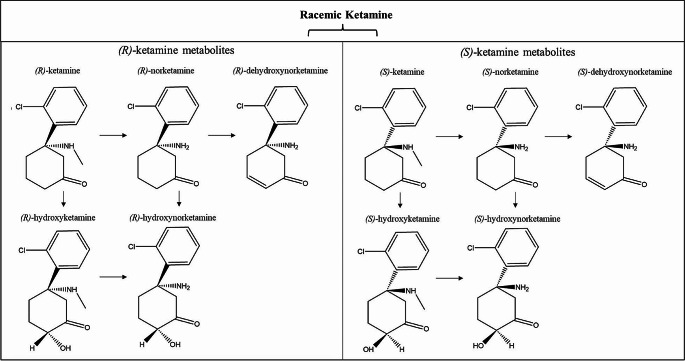
The chemical structures of (*S*)-ketamine and (*R*)-ketamine and their metabolic pathways involve stereoselective metabolism by P450 liver enzymes. Initially, both enantiomers undergo nitrogen-mediated demethylation, converting them to their norketamine (NK) forms. Further metabolism results in either dehydroxynorketamine (DHNK) or hydroxynorketamine (HNK) through hydroxylation. Each enantiomer can also be metabolized to hydroxyketamine (HK), serving as another intermediary step towards HNK. Adapted from [18]

Initial studies of ketamine’s neuropsychiatric effects focused on IV racemic (*R*,* S*)-ketamine, which is composed of two optical enantiomers rotated around an asymmetric second carbon of a cyclohexanone radical: (*S+*) and (*R-*)-ketamine (Fig. 1). However, each enantiomer holds a distinct pharmacokinetic profile. (*S*)-ketamine is demethylated at a significantly higher rate than (*R*)-ketamine or racemic ketamine [15], lending it a wider distribution pattern than its counterparts. While the exact reason underlying this pharmacokinetic difference is unknown, research has suggested that CYP3A5 (one of the main liver enzymes responsible for the demethylation of ketamine) is able to demethylate (*S*)-ketamine far more rapidly than (*R*)-ketamine [16]. As a racemic mixture, (*R*)-ketamine is also able to inhibit the metabolism of (*S*)-ketamine [17]. Although clinical enantiomer-specific research has been limited, preclinical models have found that prophylactic effects against chronic unpredictable mild stress and lipopolysaccharide injection differ between the enantiomers, and that there are additional differences between both their rapid-acting and sustained antidepressant-like effects [18]. Despite great promise in preclinical models, (*R*)-ketamine has yet to demonstrate significant clinical effects in Phase 2 clinical trials [19, 20]. In contrast, the success of esketamine in preclinical models has translated into observed clinical effects to address symptoms of TRD, leading to approvals of the intranasally-administered Spravato by the FDA and European Medicines Agency (EMA). In turn, the successful translation to approved treatment has further energized mechanistic studies and the search for putative biomarkers of antidepressant response to esketamine.

Although animal models have been extremely useful in elucidating the molecular mechanisms of ketamine and its enantiomers [18], a full description of the preclinical evidence is beyond the scope of this current review. Nevertheless, considerable research into esketamine’s molecular mechanisms is germane to this focused discussion. Generally, both (*S*)- and (*R*)-ketamine seem to enhance the likelihood of glutamate release post-administration, thereby increasing α-amino-3-hydroxy-5-methyl-4-isoxazole propionic acid receptor (AMPAR) activity [6]. This increase in AMPAR activity triggers downstream effects, such as increases in brain-derived neurotrophic factor (BDNF) release and activation of mechanistic target of rapamycin complex 1 (mTORC1) or tropomyosin receptor kinase B (TrkB) signaling pathways [21]. Despite this consistent observation, uncertainty persists about the exact mechanisms through which ketamine and its enantiomers promote this glutamate surge. One prominent hypothesis is that ketamine selectively inhibits GluN2b-containing NMDARs, which are predominantly found on gamma aminobutyric acid (GABA)-ergic interneurons. This inhibition leads to the disinhibition of cortical pyramidal neurons, resulting in a glutamate influx into the synaptic cleft, where it binds to AMPARs and initiates downstream signaling cascades [22, 23]. Interestingly, while (*S*)-ketamine (Ki = 465 nM) shows high affinity for NMDARs, (*R*)-ketamine (Ki = 1340 nM) exhibits considerably lower potency. Another potential mechanism by which ketamine might facilitate glutamate release is through blockade of extrasynaptic NMDARs, leading to the dephosphorylation of eukaryotic elongation factor 2, which in turn disinhibits BDNF release. This disinhibition results in the increased insertion of GluA1 and GluA2 into the post-synaptic membrane, thereby inducing homeostatic scaling of the synapse and enhancing synaptic AMPAR signaling [24–26].

Repeated intraperitoneal administration of (*S*)-ketamine has also been found to counteract stress-induced deficits in behavior, neuronal structure, and hippocampal long-term potentiation. This effect is achieved through Rac1-mediated synaptic plasticity, which upregulates the expression of GluA1, PSD-95, and Synapsin I [27]. Other studies have indicated that (*S*)-ketamine might preferentially activate mu-opioid and kappa-opioid receptors more than (*R*)-ketamine, potentially contributing to its risk of misuse [28, 29] as well as its antidepressant effects [30–33]. Another mechanism critical for ketamine’s antidepressant effects is suppression of NMDAR-dependent burst firing in the lateral habenula (LHb). The LHb, generally known as an “anti-reward” center, is suppressed by ketamine, which contributes to its anti-anhedonic effects [34]. A single systemic injection of racemic ketamine is capable of blocking NMDAR activity in the LHb for up to 24 h, in contrast to its much shorter actions observed in other brain regions. Through neural activity-mediated manipulation of the rate of untrapping, researchers were able to prolong or shorten ketamine’s antidepressant-like effects [35]. In this context, while preclinical investigations have yielded significant insights into the molecular mechanisms of novel therapeutics, understanding the actions of these agents in clinical populations is particularly important. For example, one clinical study found that the mTORC1 inhibitor rapamycin extended, rather than blocked, ketamine’s antidepressant effects [36]. While this does not negate other preclinical findings, it demonstrates the importance of considering clinical translation within any attempt to evaluate pharmacological actions.

Given the measured pace of neuropsychiatric drug development, knowledge of potential clinical biomarkers for future adaptive trial designs would be invaluable. This review seeks to synthesize the evidence across clinical trials in order to assess the impact of esketamine on proposed biomarkers of depression and treatment response. Because this is a nascent field, there are areas in which racemic ketamine’s effects have been extrapolated to fill the gaps in our understanding. Further work is required to elucidate the putative biomarker effects that are specific to or can be attributed to esketamine and those that cannot.

### Clinical updates

Esketamine’s first clinical approval in 2019 in an IN spray formulation (Spravato, Janssen Pharmaceuticals, Inc.) for TRD heralded what seemed to be the realization of decades of anticipation; in stark contrast to previous generations of pharmacological antidepressant treatment, esketamine had a dramatically short latency to response of hours or days rather than the weeks or longer timeline that occurs with conventional antidepressants. In 2020, its approval was expanded to treat individuals with major depressive disorder (MDD) with suicidal ideation or behavior. However, in practice, several barriers remain to its widespread use, including limitations to availability, access, and affordability [37]. In the US, esketamine includes a Boxed Warning highlighting the risk of suicidal thoughts and behaviors in pediatric and young adult patients taking antidepressants. As a result, a Risk Evaluation and Mitigation Strategy (REMS) was put into place to mitigate the risks of serious adverse outcomes resulting from sedation, dissociation, respiratory depression, or potential abuse or misuse, and the medication is available to patients only through healthcare providers at REMS-certified treatment centers. Similar barriers vary by country. For example, esketamine is listed as a Schedule 2 Controlled Drug in the UK, where it is subject to the complete Controlled Drug requirements related to prescribing, safe custody, and maintenance of a register [38]. In New Zealand, esketamine is classified as a Class C Controlled Drug (moderate risk) with specific prescribing and dispensing regulations [39], while in Australia it is controlled as a Schedule 8 drug, like ketamine and many narcotics, making it available with restrictions to reduce abuse, misuse, and dependence [40]. Even across EMA member states, the status of special and restricted medical prescriptions are categorized at the member state level [41]. Additional caveats for patients are that both approved indications require concurrent treatment with an oral antidepressant medication; prescribers must monitor patients for at least two hours after administration; and the multiple device activations needed to attain the therapeutic dose may make delivery cumbersome (for instance, in order to use the currently approved device, an individual prescribed the maximum 84 mg dose would need to receive six doses total administered across three nasal spray devices).

Although expert opinion and consensus statements recommend caution and use of ketamine and esketamine only after most other pharmacological approaches have been exhausted [42, 43], the use of both agents continues to grow. According to IQVIA National Prescription Audit, total prescriptions for esketamine dispensed in the US were approximately 87,274 in 2020, 145,475 in 2021, and 235,906 in 2022 [44]. A variety of delivery approaches also continue to be explored for ketamine and related molecules, including esketamine, in the context of other indications such as postpartum depression, anorexia nervosa, and substance use disorders [45–49]. Ketamine is widely available via mail-order from telehealth services as sublingual troches, inviting further questions about the balance between regulatory and patient safety landscapes versus practicality, patient comfort, and access to treatment [50].

The first evidence that ketamine could be safely and effectively delivered intranasally was a randomized, crossover, double-blind ketamine study of 18 patients [51]. The potential benefits of IN delivery include greater availability in a wider range of treatment settings, the need for fewer support services, and less monitoring and potential patient discomfort. IN ketamine had already been in use to treat headache [52] and chronic pain [53, 54] in ambulatory patients. Although few direct comparisons have been done, the magnitude of esketamine’s antidepressant benefit has generally been shown to be similar to racemic ketamine infusion [55, 56]. One non-inferiority trial found that IV esketamine (0.25 mg/kg) was non-inferior to racemic ketamine (0.5 mg/kg) at 24 h. At seven days there seemed to be a slight increase in remission rates in the racemic ketamine group compared to esketamine, but this was not statistically significant [57]. A retrospective comparative analysis of 210 patients found no differences in suicidality items, or in remission and response rates, between the two agents, although the study was unrandomized and unblinded [58]. An observational study of 62 adults in real-world clinical settings also found no difference in response or remission but did report a faster time to remission with IV racemic ketamine compared to IN esketamine [59]. When pooled data from three of these studies comparing IV ketamine to IN esketamine were combined, similar response and remission rates were observed, but response time for IV ketamine was quicker [60]. A systematic review of IN esketamine versus IV racemic ketamine demonstrated a similar number-needed-to-treat (NNT) for both treatments, with NNTs of two at one day and 11 at four weeks for esketamine, and NNTs of three at one to seven days and nine at four weeks [61]. In terms of dosing, IV esketamine at 0.2 mg/kg was superior to placebo in one trial [62], while IV racemic ketamine at 0.2 mg/kg was not superior in two other randomized trials, potentially due to low-dose administration not allowing for proper metabolite concentrations [63, 64]. Collectively, this suggests that there is insufficient evidence from which to strongly recommend one treatment modality over another from a clinical efficacy standpoint.

### Landmark intranasal esketamine clinical studies

Development of ketamine’s (*S*)-enantiomer proceeded based on its reportedly higher affinity for the NMDAR than (*R*)-ketamine [65]. One of the first studies to assess the efficacy and safety of IN esketamine for TRD was a Phase 2, double-blind, placebo-controlled study across multiple outpatient referral centers; 67 individuals with TRD (defined as inadequate response to two or more antidepressants) were randomized to receive placebo or esketamine at 28, 56, or 84 mg twice weekly while continuing their current antidepressant regimen [66]. Most of those who received placebo were re-randomized to the four treatment arms. All three doses of esketamine were superior to placebo in reducing Montgomery-Asberg Depression Rating Scale (MADRS) total score after one week in a dose-dependent manner; this effect was sustained for up to nine weeks after decreasing frequency during the open-label phase to biweekly.

Another double-blind, multicenter, placebo-controlled study randomized 68 participants to receive IN esketamine alongside standard-of-care treatment. With twice weekly dosing, IN esketamine reduced MADRS scores at 4 h and 24 h after the initial dose, but not at 25 days [67]. The MADRS suicidal thoughts score at 4 h was also significantly reduced, but not at later timepoints, and there was no reduction in clinician global judgment of suicide risk at any timepoint. Dissociative symptoms, which peaked at 40 min after dosing and resolved by two hours, attenuated with repeated dosing over time. Three participants required a dose reduction due to intolerance, while five had adverse events leading to early discontinuation (agitation, aggression, unpleasant taste, and ventricular extrasystoles in one participant each, and dizziness, dyspnea, and nausea in one participant).

The TRANSFORM-1 trial, a Phase 3, double-blind, multicenter study, enrolled 346 adults with moderate-to-severe depression who had not responded to at least two antidepressant trials during the current depressive episode [68]. Participants were randomized to 56 or 84 mg of IN esketamine or placebo spray twice weekly alongside a newly initiated open-label oral medication. After four weeks, the combined esketamine groups together demonstrated a clinically meaningful effect versus placebo, although the primary endpoint specifically comparing 84 mg esketamine versus placebo was not met. The positive finding was confirmed in another Phase 3 efficacy trial (*n* = 197), where change in MADRS score at day 28 was significantly greater in those receiving flexibly dosed IN esketamine versus placebo [69].

As mentioned previously, the IN esketamine formulation Spravato was granted FDA and EMA approval in 2019 [70]. Subsequent FDA approval in July 2020 of esketamine to treat MDD with acute suicidal ideation or behavior was based on two identical Phase 3 trials. ASPIRE I, which was conducted between June 2017 and December 2018, assessed the effects of IN esketamine (84 mg, twice weekly for four weeks) or placebo on 226 participants with MDD and active suicidal ideation with intent. IN esketamine was given alongside comprehensive standard-of-care treatment, including initial psychiatric hospitalization and optimization or initiation of oral antidepressants. The primary endpoint was MADRS score 24 h post-initial dose, and significant decreases were observed for esketamine plus standard-of-care compared to placebo plus standard-of-care. These differences were also present at four hours post-dose and sustained through day 25 of treatment. The most common adverse events included dizziness, headache, dissociation, somnolence, and nausea. Depression- and suicide-related adverse events, which were considered unrelated to esketamine treatment, included three attempted and one completed suicide in those who received esketamine during the follow-up phase, and two attempted suicides in the placebo group [71]. ASPIRE II (*n* = 230), which used an identical study design, similarly found significant reductions in MADRS score compared to placebo at 24 h, which continued to favor esketamine at timepoints through day 25. Both groups experienced significant reductions of severity of suicidality and were not significantly different. The adverse events profile was similar to the first trial, with the addition of dysgeusia and paresthesia amongst the common complaints for those who received esketamine [72]. The ESCAPE-TRD open-label, randomized, multi-site trial compared esketamine to extended-release quetiapine, which is an approved adjunctive treatment for TRD; both agents were additionally compared to treatment with a selective serotonin reuptake inhibitor (SSRI) or serotonin-norepinephrine reuptake inhibitor (SNRI) during an initial eight-week treatment period as well as during a subsequent 24-week maintenance phase [73]. Of the 776 randomized patients, more patients in the esketamine group achieved remission at week 8. After 32 weeks of maintenance and follow-up, esketamine was favored with regard to sustained remission rates, response rates, and change in MADRS score from baseline.

Given that FDA approval was granted in 2019, data are beginning to accumulate with regard to esketamine’s long-term maintenance effects. For instance, post-hoc results from the SUSTAIN-2 trial suggest efficacy in adjusting treatment frequency in accordance with depressive symptoms. After the initial phase of twice-weekly dosage, participants received esketamine once per week for the following four weeks; 76% maintained clinical benefit or had further reductions in depressive symptom scores. Antidepressant response continued to be maintained in a similar proportion of individuals when the esketamine dose was further reduced to every other week; furthermore, in 90% of those who worsened, re-increasing the dose to once a week improved clinical benefit or stabilized mood ratings [74]. In addition, interim results from SUSTAIN-3, a long-term (2 + years) open-label study (*n* = 1148), suggest that esketamine has antidepressant effects that last throughout the optimization and maintenance periods, with persistent reductions observed in MADRS scores [75]. Roughly 31% of the participants in the SUSTAIN-3 study discontinued treatment during the optimization/maintenance phase for a variety of reasons, with no differences in discontinuation rates observed across length of time in the study. In addition, a SUSTAIN-3 subgroup analysis in those who experienced a second induction and maintenance period after relapse found remission rates around 61% throughout the second optimization/maintenance period, suggesting that potential benefits are associated with a second induction period [76].

Based on these seminal studies, current guidelines recommend co-administration of a traditional oral antidepressant with esketamine. Dosing for IN ketamine is typically 56 mg on day 1, with subsequent dosing of 56 mg or 84 mg per insufflation. The recommended frequency is twice a week for four weeks, followed by once a week for four weeks, then once every one to two weeks thereafter. Monitoring is recommended for at least two hours after administration, which may require reporting of vital signs and return to baseline functioning to a centralized monitoring system.

### Putative clinical biomarkers research

#### Blood, saliva, and other measures

To date, few clinical studies have examined biomarkers associated with esketamine; the current literature is summarized in Table 1. For example, one systematic review published in 2022 found only two previous studies of blood-based putative biomarkers examining response to esketamine [77]. A genome-wide association study (GWAS) of the participants in the SUSTAIN-2 and TRANSFORM-3 Phase 3 trials found significant associations between clinical response to esketamine and interleukin-1 receptor associated kinase-3 (*IRAK3*). NME/NM23 family member 7 (*NME7*) was also associated with change in MADRS score as determined by gene-level association analysis. *NME7* is a functional component of the γ-tubulin ring complex and involved in microtubule organization and cell division in various tissues [78], though little is known about its relationship to depressive symptoms. Pathway enrichment analyses also suggested that glucocorticoid metabolic processes (*p* = 3.53 × 10^− 5^) and neuronal action potential (*p* = 0.0001) were associated with change in MADRS score after treatment. The genetic loading for depressive symptoms was most strongly associated with esketamine efficacy as identified by polygenic risk score analysis, though this did not reach study-wide significance after multiple testing corrections (*p* = 0.001, standardized coefficient β=− 3.1, SE = 0.9) [79]. In a metabolomic profile, decreases in tryptophan metabolites (indole-3-lactate and indole-3-acetate) were observed two hours post-esketamine infusion, potentially implicating kynurenine signaling and the gut microbiome in esketamine’s mechanism of action [80]. Finally, a meta-analysis of metabolomic profiles after ketamine and esketamine treatment found changes in mitochondrial function and kynurenine signaling but emphasized the importance of considering time after administration of ketamine in analyses [81].

**Table 1:** Biomarkers from clinical studies of esketamine

Biomarker Type	Disorder	Dose and Administration	Study Type	Clinical Results	Biomarker Results	Reference
Blood	Breast cancer with mild/moderate depression	0.125 mg/kg intravenous esketamine before surgery	RCT (*n* = 303)	Significant decreases in HAMD-17 scores three days to one month after surgery compared to racemic ketamine and placebo	Increased serum levels of BDNF and 5-HT	[82]
Blood	Laparoscopic radical hysterectomy	0.2–0.5 mg/kg intravenous esketamine, 0.5 mg/kg ketamine	RCT (*n* = 417)	0.5 mg/kg of esketamine significantly decreased VAS and HAMD-17 scores	Significant increases in serum BDNF and 5-HT	[83]
Blood	Non-cardiac thoracic surgery patients	0.2–0.5 mg/kg intravenous esketamine	RCT (*n* = 129)	0.5 mg/kg of esketamine significantly decreased symptoms of post-operative anxiety and depression	Significant increases in serum BDNF, and decreases in S100β and IL-6.	[84]
Blood	TRD	Intravenous esketamine (0.20 or 0.40 mg/kg), ketamine (0.5 mg/kg), or placebo	RCT (*n* = 53)	Mean % MADRS change was -47.73% for those receiving esketamine and − 29.84% for those receiving ketamine	Changes in tryptophan metabolites were found 2 h after esketamine infusion	[80]
Genetic	TRD	Intranasal esketamine, flexible dose	Samples from two RCTs: SUSTAIN-2 (*n* = 598) and TRANSFORM-3 (*n* = 95)	SUSTAIN-2 and TRANSFORM-3 clinical data published elsewhere	Associations between IRAK3 and NME7 and esketamine efficacy. Polygenic score analysis demonstrated genetic loading for depressive symptoms most associated with esketamine efficacy.	[79]
Imaging	Gynecological surgery	0.125 mg/kg intravenous esketamine before sevoflurane anesthesia	RCT (*n* = 44)	No difference in cognition or anesthesia emergence time.	Decreased slow, delta, and alpha waves and increased beta-gamma bands in prefrontal EEG.	[85]
Imaging	HV	0.12 mg/kg initiation, 0.25 mg/kg continuous intravenous esketamine	Open-label (*n* = 23)	No clinical measures recorded.	Stronger pgACC activation during esketamine infusion was associated with glutamine concentrations. Reduced functional connectivity between the pgACC and aMCC was also associated with reduced glutamine.	[86]
Imaging	HV	0.1 mg/kg initiation, 0.015625 mg/kg min continuous intravenous esketamine	RCT (*n* = 23)	No clinical measures recorded.	Esketamine led to downregulation of functional connectivity in the mPFC and an increase in the intraparietal cortices. A shift from slow-wave to gamma wave frequencies was also observed after esketamine.	[87]
Imaging	HV	0.11 mg/kg initiation, 0.12 mg/kg maintenance intravenous esketamine	RCT (*n* = 31)	N/A	Hippocampal subfield volumes significantly increased post-esketamine treatment.	[88]
Imaging	HV	0.33 mg/kg intravenous esketamine	RCT (*n* = 35)	Changes in connectivity were not associated with any psychotomimetic effects	Immediate increase in rsFC from pgACC to mPFC that was associated with increased glutamate levels. A delayed decrease in rsFC from the pgACC to the inferior parietal lobe and dlPFC.	[89]
Imaging	HV	0.11 mg/kg initiation, 0.12 mg/kg maintenance intravenous esketamine	RCT (*n* = 35)	Esketamine significantly upregulated the experience of unity and disembodiment, as measured by the 5D-ASC	Negative correlations were found between PCC cortical thickness and measures of disembodiment. pgACC thickness was not associated with any measures of sense of self.	[90]
Imaging	HV	0.375 mg/kg intravenous esketamine	Uncontrolled pre-post intervention (*n* = 25)	Increases in PANSS scores and decreases in positive affect after esketamine administration.	The ACC, mPFC, insula, left accumbens, and left ventral caudate had signifcant increases in rCBF, which was associated with increased glutamate levels.	[91]
Imaging	TRD	0.25 mg/kg intravenous esketamine, 0.5 mg/kg intravenous ketamine	(*n* = 21)	91% of participants had reduced MADRS and/or HAMD scores at 24 h post-infusion, with mean symptom improvement at 29.81%	Increased cerebral blood flow to thalamus after ketamine/esketamine treatment (note: esketamine not separated for analyses)	[86]
Imaging	TRD	0.25 mg/kg intravenous esketamine, 0.5 mg/kg intravenous ketamine	(*n* = 24)	92% of participants had reduced MADRS scores, with 33% classified as responders	Increased PFC-sgACC resting state connectivity after ketamine/esketamine treatment (note: esketamine not separated for analyses)	[92]
Imaging	TRD	0.25 mg/kg intravenous esketamine, 0.5 mg/kg intravenous ketamine	Naturalistic, open-label (*n* = 33)	30.3% of participants were responders at 24 h	Baseline rACC volume predicted treatment response at 24 h.	[93]
Imaging	TRD	0.25 mg/kg intravenous esketamine, 0.5 mg/kg intravenous ketamine	RCT (*n* = 24)	Mean clinical improvement was 22.6% on the BDI	pgACC activity during an emotional stimulation task predicted treatment response at 24 h, and increased glutamate at 24 h was associated with better clinical outcomes.	[94]
Other	iPSC-derived dopaminergic neurons	0.1–30 μm esketamine	Other	N/A	Esketamine quickly entered the ER and may interact with organelle channels, receptors, or transporters	[95]
Other	Swiss Target Prediction Software	N/A	Other	N/A	Both ketamine enantiomers were predicted to interact with NMDARs and phosphodiesterases 3 A, 5 A, and 7 A, while esketamine was uniquely predicted to interact with the GABAergic system	[96]

**Abbreviations**: 5D-ASC: 5-Dimensional Altered States of Consciousness Rating Scale; 5-HT: serotonin; ACC: anterior cingulate cortex; BDI: Beck Depression Inventory; BDNF: brain-derived neurotrophic factor; EEG: electroencephalogram; ER: endoplasmic reticulum; GABA: gamma aminobutyric acid; HAM-D: Hamilton Depression Rating Scale; HV: healthy volunteer; IL-6: interleukin-6; iPSC: induced pluripotent stem cells; aMCC: anterior midcingulate cortex; MADRS: Montgomery-Asberg Depression Rating Scale; mPFC: medial prefrontal cortex; NMDAR: N-methyl-D-aspartate receptor; PANSS: Positive and Negative Syndrome Scale for Schizophrenia; PCC: posterior cingulate cortex; pgACC: perigenual anterior cingulate cortex; rACC: rostral anterior cingulate cortex; rCBF: regional cerebral blood flow; RCT: randomized controlled trial; rsFC: resting-state functional connectivity; sgACC: subgenual anterior cingulate cortex; TRD: treatment-resistant depression; VAS: Visual Analogue Scale

Another meta-analysis demonstrated significant, but transient and relatively small, effects for both ketamine and esketamine on increases in systolic and diastolic blood pressure, as well as heart rate [97]; no significant differences were observed between ketamine and esketamine. In healthy volunteers, esketamine almost doubled production of saliva and plasma cortisol expression over a three-hour period in a manner consistent with circadian rhythms [98]. Future research is needed to correlate enantiomer-specific expression after racemic ketamine administration with biological and clinical measures using convenient tools such as chiral liquid chromatography/tandem mass spectrometry (LC-MS/MS) assays [99, 100].

#### Neuroimaging and electrophysiology

Increases in frontoparietal gamma power, functional connectivity within the prefrontal cortex (PFC), and striatum activation have all been previously associated with magnitude of antidepressant response to ketamine [101–103]. While most research in this area has focused on racemic ketamine, two studies that examined both ketamine and esketamine in their analyses found associations between clinical response and increased thalamic blood flow through perfusion MRI [86] and increases in resting-state functional connectivity between the PFC and subgenual cingulate [92]. Other studies found that greater baseline volume of the bilateral rostral anterior cingulate cortex (ACC) [93], greater pregenual ACC (pgACC) activity during emotional stimulation, and increased glutamate levels at 24 h [94] all significantly predicted treatment response to both ketamine and esketamine. Another study found that low-dose esketamine administered during anesthesia had no impact on electromyography measures [104].

In healthy volunteers, esketamine led to immediate upregulation of resting-state functional connectivity (rsFC) to the dorsomedial PFC, followed by a decreased connectivity of the pgACC to the parietal lobe and dorsolateral PFC (dlPFC). The immediate increases in rsFC also correlated with increased MRS glutamate levels, as estimated by a neurometabolite fitted spectral curve, in the pgACC [89]. Additional neuroimaging research in healthy volunteers found that esketamine may increase hippocampal subfield volumes [88], increase resting cerebral blood flow [91], lead to changes in slow and fast (gamma) wave frequencies [87], and alter pgACC to medial ACC connectivity [86]. pgACC cortical thickness has also been found to be negatively correlated with disembodiment ratings after esketamine administration [90]. However, results from healthy volunteers should be interpreted with caution, given that ketamine administration is also commonly used as a model for schizophrenia and neurocognitive impairment within healthy populations [105]. Further research is needed within TRD populations, and multiple clinical trials using a variety of neuroimaging techniques are currently recruiting TRD participants and healthy volunteers to further understand brain network activity after esketamine administration (NCT06012916 (K-BRAINED), NCT04587778, NCT06002100, NCT05137938).

#### Beyond clinical trials

Research in this area is understandably limited by what can be accomplished within human populations. Within the past decade, novel methods of assessing human neurobiology have been developed, including in vitro induced pluripotent stem cell (iPSC) models, machine learning, and network analyses. The genetically encoded biosensors iSKetSnFR1 and iSKetSnFR2 fluorescently respond to the presence of esketamine in different cellular compartments and can be used in in vitro models. In iPSC-derived dopaminergic neurons, esketamine rapidly enters the endoplasmic reticulum, suggesting that organellar ion channels, receptors, and transporters may be a potential target of esketamine’s antidepressant effects [95]. Network pharmacology analysis using the Swiss Target Prediction software predicted esketamine to uniquely interact with the GABAergic system, identifying main targets with gene ontology and KEGG enrichment analysis [96]. Additional insights into esktamine’s effects on clinical populations will likely be gained as computational modeling and in vitro models improve.

#### Putative biomarkers of esketamine in other indications

The use of subanesthetic esketamine in other contexts can also provide useful insights into the biological mechanisms of treatment response. For instance, prefrontal EEG revealed that subanesthetic esketamine decreased the power of slow, delta, and alpha waves while increasing the power of beta-gamma bands during sevofluorane anesthesia, though differences in cognition and emergence time were not noted in comparison to placebo [85].

In breast cancer patients, pre-treatment with esketamine before surgery significantly increased serum levels of BDNF and serotonin; these levels, in turn, were negatively correlated with significant decreases in post-operative depression compared to both ketamine and placebo [82]. Ongoing trials seek to assess the effects of an intraoperative sub-anesthetic dose of esketamine on depression in women undergoing radical mastectomies [106] and on post-operative delirium in the elderly undergoing non-cardiac thoracic surgery (NCT05242692). Another study found that low-dose esketamine (0.5 mg/kg) also effectively reduced post-operative anxiety and depression in those undergoing thoracic surgery, and that symptom decreases were associated with increased serum BDNF levels and decreased S100β and interleukin-6 levels [84]. Similarly, 0.5 mg/kg of esketamine increased serum BDNF and serotonin levels for one to three days post-hysterectomy, and these increases were associated with significantly decreased Visual Analogue Scale (VAS) and Hamilton Depression Rating Scale (HAMD-17) scores [83].

#### Adverse events and risk of misuse

While esketamine has demonstrated significant clinical efficacy in TRD, its clinical use is not without risks. Concomitant use of benzodiazepines may interfere with response to ketamine, leading to the general practice of withholding them at least 24 h prior to ketamine infusion, a guideline that is also usually applied to IN esketamine administration [107–109]. In a seminal RCT (*n* = 67) followed by a nine-week open-label phase, adverse events such as syncope, headache, dissociation, and ectopic pregnancy led to study discontinuation in one participant each [66]. Double-blind RCTs comparing IN esketamine to placebo administered alongside standard care found that nausea (~ 27–40%), dizziness (~ 12–22%), dissociation (~ 12–26%), unpleasant taste (14–16%), and headache (~ 20–26%) were the most commonly reported side effects [67, 68]. It should also be noted that long-term esketamine use may increase risk of urinary tract symptoms, though this has not been reported from administration following clinical guidelines [110].

Although there has also been concern regarding the impact of ketamine on cognition, most research in individuals with TRD suggests that ketamine/esketamine administration leads to overall improvements in cognition-related tasks such as working memory, processing speed, and cognitive flexibility, among others [111]. Repeated dose esketamine in 51 adolescents with MDD and suicidal ideation also led to significant improvements in processing speed and working memory after 12 days [112]. Another small study in eight patients with TRD also found long-term improvements in cognition after three months of treatment with IN esketamine [113]. Another study found that a single dose of esketamine did not impair driving performance eight hours after administration compared to placebo, in contrast to a positive control of an impairing dose of mirtazapine [114]. Longer-term studies, larger sample sizes, and the inclusion of careful controls are necessary to fully determine any potential impact of esketamine on cognition.

Although patient monitoring during esketamine administration can be burdensome, new technologies like the “MindMed Session Monitoring System” have been developed to alleviate this strain. This system, tested for use with Spravato administration, includes heart rate, motion, audio, and additional activity data that provide passive physiological monitoring of patients [115]. The use of these and similar systems, or even widespread technology such as fitness trackers, smart watches, or other wearables, will enable the future collection of additional empirical data regarding esketamine’s impact on the body.

In addition to administration-related adverse events, esketamine also carries a risk of potential misuse liability, even though delivery application devices were designed with this risk in mind. Recent preclinical studies suggest that esketamine may preferentially activate mu-opioid receptors, suggesting it may have stronger reinforcement properties than racemic or (*R*)-ketamine [29]. An analysis of esketamine cases extracted from the FDA Adverse Event Reporting System and EMA EudraVigilance database found cases that may reflect abuse potential, as determined by the presence of qualities such as “feeling drunk”, “hallucination”, and “derealization” as well as preferred terms such as “withdrawal syndrome”, “dependence”, and “off-label use”, among others [116]. Despite these findings, little evidence suggests that esketamine administered according to clinical guidelines leads to substance misuse [58]. However, caution is necessary in real-world settings where off-label prescribing and improper supervision could have potentially grievous consequences.

## Conclusions

Esketamine is the first widely available agent to emerge from the ketamine-inspired revolution in psychiatric pharmacotherapy. The evidence demonstrates that esketamine is a novel clinical treatment option addressing TRD and suicidality and a unique tool to probe for putative biomarkers of antidepressant response and mechanisms of action; indeed, the limited existing evidence suggests that esketamine seems to have efficacy on par with that of racemic ketamine, although more direct, designed comparisons are needed to test this. However, the study of biomarkers associated with response to esketamine in clinical research is still preliminary, as few studies have sought to identify potential key components of antidepressant response. Significant associations with genes such as *IRAK3* and *NME7* suggest that inflammation may play a role in esketamine’s rapid-acting antidepressant effects, though future research is necessary given the mixed literature to date on ketamine’s anti- versus pro-inflammatory properties [117]. Metabolomic profiles also suggest that the gut microbiome and mitochondrial function may play a role in mediating esketamine’s effects, although that research is preliminary [80]. Transient effects on cortisol levels, blood pressure, and heart rate could also contribute to both esketamine’s side effects and treatment profile. Clinical neuroimaging and electrophysiological studies to date have been more common than blood-based biomarker studies; these seem to consistently implicate the PFC, striatum, and ACC in esketamine’s rapid and longer-lasting antidepressant effects. Novel methodologies such as iPSC models and network pharmacology may also provide new insight into esketamine’s mechanisms. Finally, clinical research into other indications has also provided useful insight into potential biological mechanisms, including demonstrating promise in perioperative settings for reducing depression and anxiety, with corresponding changes in biomarkers like BDNF and serotonin. Future clinical research should prioritize genomic, proteomic, and brain connectivity biomarkers in TRD populations to fully elucidate the mechanisms underlying esketamine’s therapeutic effects.

## References

[1] Mion G (2017) History of anaesthesia: the ketamine story – past, present and future. Eur J Anaesthesiol 34:571–575 28731926 10.1097/EJA.0000000000000638

[2] Nowak G, Trullas R, Layer RT, Skolnick P, Paul IA (1993) Adaptive changes in the n-methyl-d-aspartate receptor complex after chronic treatment with imipramine and 1-aminocyclopropanecarboxylic acid. J Pharmacol Exp Ther 265:1380–1386 8099620

[3] Berman RM, Cappiello A, Anand A, Oren DA, Heninger GR, Charney DS, Krystal JH (2000) Antidepressant effects of ketamine in depressed patients. Biol Psychiatry 47:351–354 10686270 10.1016/s0006-3223(99)00230-9

[4] Zarate CA Jr., Brutsche NE, Ibrahim L, Franco-Chaves J, Diazgranados N, Cravchik A, Selter J, Marquardt CA, Liberty V, Luckenbaugh DA (2012) Replication of ketamine’s antidepressant efficacy in bipolar depression: a randomized controlled add-on trial. Biol Psychiatry 71:939–946 22297150 10.1016/j.biopsych.2011.12.010 PMC3343177

[5] Zarate CA Jr., Singh JB, Carlson PJ, Brutsche NE, Ameli R, Luckenbaugh DA, Charney DS, Manji HK (2006) A randomized trial of an n-methyl-d-aspartate antagonist in treatment-resistant major depression. Arch Gen Psychiatry 63:856–864 16894061 10.1001/archpsyc.63.8.856

[6] Zanos P, Gould TD (2018) Mechanisms of ketamine action as an antidepressant. Mol Psychiatry 23:801–811 29532791 10.1038/mp.2017.255 PMC5999402

[7] Herd DW, Anderson BJ, Keene NA, Holford NH (2008) Investigating the pharmacodynamics of ketamine in children. Paediatr Anaesth 18:36–42 18095964 10.1111/j.1460-9592.2007.02384.x

[8] MacDonald J, Miljkovic Z, Pennefather P (1987) Use-dependent block of excitatory amino acid currents in cultured neurons by ketamine. J Neurophysiol 58:251–266 2443623 10.1152/jn.1987.58.2.251

[9] Huettner JE, Bean BP (1988) Block of n-methyl-d-aspartate-activated current by the anticonvulsant mk-801: selective binding to open channels. Proc Natl Acad Sci 85:1307–1311 2448800 10.1073/pnas.85.4.1307 PMC279756

[10] Desta Z, Moaddel R, Ogburn ET, Xu C, Ramamoorthy A, Venkata SLV, Sanghvi M, Goldberg ME, Torjman MC, Wainer IW (2012) Stereoselective and regiospecific hydroxylation of ketamine and norketamine. Xenobiotica 42:1076–1087 22612619 10.3109/00498254.2012.685777 PMC3426663

[11] Rao LK, Flaker AM, Friedel CC, Kharasch ED (2016) Role of cytochrome p4502b6 polymorphisms in ketamine metabolism and clearance. Anesthesiology 125:1103–1112 27763887 10.1097/ALN.0000000000001392

[12] Domino EF, Domino SE, Smith RE, Domino LE, Goulet JR, Domino KE, Zsigmond EK (1984) Ketamine kinetics in unmedicated and diazepam-premedicated subjects. Clin Pharmacol Ther 36:645–653 6488686 10.1038/clpt.1984.235

[13] Schüttler J, Stanski DR, White PF, Trevor AJ, Horai Y, Verotta D, Sheiner LB (1987) Pharmacodynamic modeling of the eeg effects of ketamine and its enantiomers in man. J Pharmacokinet Biopharm 15:241–253 3668802 10.1007/BF01066320

[14] Sigtermans M, Dahan A, Mooren R, Bauer M, Kest B, Sarton E, Olofsen E (2009) S (+)-ketamine effect on experimental pain and cardiac output: a population pharmacokinetic-pharmacodynamic modeling study in healthy volunteers. Anesthesiology 111:892–903 19741495 10.1097/ALN.0b013e3181b437b1

[15] Geisslinger G, Hering W, Kamp H, Vollmers K (1995) Pharmacokinetics of ketamine enantiomers. Br J Anaesth 75:506–507 7488510 10.1093/bja/75.4.506

[16] Portmann S, Kwan HY, Theurillat R, Schmitz A, Mevissen M, Thormann W (2010) Enantioselective capillary electrophoresis for identification and characterization of human cytochrome p450 enzymes which metabolize ketamine and norketamine in vitro. J Chromatogr A 1217:7942–7948 20609441 10.1016/j.chroma.2010.06.028

[17] Ihmsen H, Geisslinger G, Schüttler J (2001) Stereoselective pharmacokinetics of ketamine: R (–)-ketamine inhibits the elimination of s (+)‐ketamine. Clin Pharmacol Ther 70:431–438 11719729 10.1067/mcp.2001.119722

[18] Johnston JN, Henter ID, Zarate CA Jr (2023) The antidepressant actions of ketamine and its enantiomers. Pharmacol Ther 246:108431 37146727 10.1016/j.pharmthera.2023.108431 PMC10213151

[19] Leal GC, Souza-Marques B, Mello RP, Bandeira ID, Caliman-Fontes AT, Carneiro BA, Faria-Guimarães D, Guerreiro-Costa LN, Jesus-Nunes AP, Silva SS (2023) Arketamine as adjunctive therapy for treatment-resistant depression: a placebo-controlled pilot study. J Affect Disord 330:7–15 36871913 10.1016/j.jad.2023.02.151

[20] Atai Life Sciences (2023) Atai life sciences announces results from phase 2a trial of pcn-101 (r-ketamine) for treatment-resistant depression. https://www.globenewswire.com/news-release/2023/01/06/2584334/0/en/atai-life-sciences-announces-results-from-phase-2a-trial-of-pcn-101-r-ketamine-for-treatment-resistant-depression.Html

[21] Lin P-Y, Ma ZZ, Mahgoub M, Kavalali ET, Monteggia LM (2021) A synaptic locus for trkb signaling underlying ketamine rapid antidepressant action. Cell Rep 36:109513 34407417 10.1016/j.celrep.2021.109513 PMC8404212

[22] Miller OH, Moran JT, Hall BJ (2016) Two cellular hypotheses explaining the initiation of ketamine’s antidepressant actions: direct inhibition and disinhibition. Neuropharmacology 100:17–26 26211972 10.1016/j.neuropharm.2015.07.028

[23] Widman AJ, McMahon LL (2018) Disinhibition of ca1 pyramidal cells by low-dose ketamine and other antagonists with rapid antidepressant efficacy. Proc Natl Acad Sci 115:E3007–E3016 29531088 10.1073/pnas.1718883115 PMC5879689

[24] Autry AE, Adachi M, Nosyreva E, Na ES, Los MF, Cheng P-f, Kavalali ET, Monteggia LM (2011) Nmda receptor blockade at rest triggers rapid behavioural antidepressant responses. Nature 475:91–95 21677641 10.1038/nature10130 PMC3172695

[25] Nosyreva E, Autry AE, Kavalali ET, Monteggia LM (2014) Age dependence of the rapid antidepressant and synaptic effects of acute nmda receptor blockade. Front Mol Neurosci 7:94 25520615 10.3389/fnmol.2014.00094 PMC4249453

[26] Nosyreva E, Szabla K, Autry AE, Ryazanov AG, Monteggia LM, Kavalali ET (2013) Acute suppression of spontaneous neurotransmission drives synaptic potentiation. J Neurosci 33:6990–7002 23595756 10.1523/JNEUROSCI.4998-12.2013 PMC3661220

[27] Zhu X, Zhang F, You Y, Wang H, Yuan S, Wu B, Zhu R, Liu D, Yan F, Wang Z (2023) S-ketamine exerts antidepressant effects by regulating rac1 gtpase mediated synaptic plasticity in the hippocampus of stressed rats. Cell Mol Neurobiol 43:299–314 35083636 10.1007/s10571-021-01180-6 PMC11415159

[28] Bonaventura J, Lam S, Carlton M, Boehm MA, Gomez JL, Solís O, Sánchez-Soto M, Morris PJ, Fredriksson I, Thomas CJ (2021) Pharmacological and behavioral divergence of ketamine enantiomers: implications for abuse liability. Mol Psychiatry 26:6704–6722 33859356 10.1038/s41380-021-01093-2 PMC8517038

[29] Levinstein MR, Carlton ML, Di Ianni T, Ventriglia EN, Rizzo A, Gomez JL, Budinich RC, Shaham Y, Airan RD, Zarate CA Jr (2023) Mu opioid receptor activation mediates (s)-ketamine reinforcement in rats: implications for abuse liability. Biol Psychiatry 93:1118–1126 36841701 10.1016/j.biopsych.2022.12.019 PMC11947972

[30] Levinstein MR, Michaelides M (2024) Exploring the role of mu opioid receptors in the therapeutic potential and abuse liability of (s)-ketamine. Neuropsychopharmacology 49:315–316 37438422 10.1038/s41386-023-01652-x PMC10700302

[31] Williams NR, Heifets BD, Bentzley BS, Blasey C, Sudheimer KD, Hawkins J, Lyons DM, Schatzberg AF (2019) Attenuation of antidepressant and antisuicidal effects of ketamine by opioid receptor antagonism. Mol Psychiatry 24:1779–1786 31467392 10.1038/s41380-019-0503-4

[32] Klein ME, Chandra J, Sheriff S, Malinow R (2020) Opioid system is necessary but not sufficient for antidepressive actions of ketamine in rodents. Proc Natl Acad Sci 117:2656–2662 31941713 10.1073/pnas.1916570117 PMC7007545

[33] Hailozian C, Luftig J, Liang A, Outhay M, Ullal M, Anderson ES, Kalmin M, Shoptaw S, Greenwald MK, Herring AA (2022) Synergistic effect of ketamine and buprenorphine observed in the treatment of buprenorphine precipitated opioid withdrawal in a patient with fentanyl use. J Addict Med 16:483–487 34789683 10.1097/ADM.0000000000000929

[34] Cui Y, Hu S, Hu H (2019) Lateral habenular burst firing as a target of the rapid antidepressant effects of ketamine. Trends Neurosci 42:179–191 30823984 10.1016/j.tins.2018.12.002

[35] Ma S, Chen M, Jiang Y, Xiang X, Wang S, Wu Z, Li S, Cui Y, Wang J, Zhu Y, Zhang Y, Ma H, Duan S, Li H, Yang Y, Lingle CJ, Hu H (2023) Sustained antidepressant effect of ketamine through nmdar trapping in the lhb. Nature 622:802–809 37853123 10.1038/s41586-023-06624-1 PMC10600008

[36] Abdallah CG, Averill LA, Gueorguieva R, Goktas S, Purohit P, Ranganathan M, Sherif M, Ahn K-H, D’Souza DC, Formica R (2020) Modulation of the antidepressant effects of ketamine by the mtorc1 inhibitor rapamycin. Neuropsychopharmacology 45:990–997 32092760 10.1038/s41386-020-0644-9 PMC7162891

[37] Harding L (2023) Regulating ketamine use in psychiatry. J Am Acad Psychiatry Law 51:320–325 37657825 10.29158/JAAPL.230040-23

[38] Young AH, Abdelghani M, Juruena MF, Nikolova VL, Nilforooshan R (2023) Early clinical experiences of esketamine nasal spray in the Uk in adults with treatment-resistant major depressive disorder: Advisory panel recommendations. Neuropsychiatr Dis Treat 19:433–441 36861011 10.2147/NDT.S388392 PMC9968662

[39] New Zealand Medicines and Medical Devices Safety Authority (2019) Medsafe classification database. Https://medsafe.Govt.Nz/profs/class/classintro.Asp

[40] Australian Government Department of Health and Aged Care (2024) Therapeutic goods administration. Https://www.Tga.Gov.Au, Available at

[41] European Medicines Agency (EMA) (2019) Spravato. Https://www.Ema.Europa.Eu/en/medicines/human/epar/spravato. 2024

[42] Sanacora G, Frye MA, McDonald W, Mathew SJ, Turner MS, Schatzberg AF, Summergrad P, Nemeroff CB, American Psychiatric Association (APA) Council of Research Task Force on Novel Biomarkers and Treatments (2017) A consensus statement on the use of ketamine in the treatment of mood disorders. JAMA Psychiatry 74:399–405 28249076 10.1001/jamapsychiatry.2017.0080

[43] Freedman R, Brown AS, Cannon TD, Druss BJ, Earls FJ, Escobar J, Hurd YL, Lewis DA, López-Jaramillo C, Luby J, Mayberg HS, Moffitt TE, Oquendo M, Perlis RH, Pine DS, Rush AJ, Tamminga CA, Tohen M, Vieta E, Wisner KL, Xin Y (2018) Can a framework be established for the safe use of ketamine? Am J Psychiatry 175:587–589 29656666 10.1176/appi.ajp.2018.18030290

[44] Drug Enforcement Agency (DEA) (2024) Ketamine. Https://www.Deadiversion.Usdoj.Gov/drug_chem_info/ketamine.Pdf. 2024

[45] Alipoor M, Loripoor M, Kazemi M, Farahbakhsh F, Sarkoohi A (2021) The effect of ketamine on preventing postpartum depression. J Med Life 14:87 33767791 10.25122/jml-2020-0116 PMC7982256

[46] Wang S, Deng C-M, Zeng Y, Chen X-Z, Li A-Y, Feng S-W, Xu L-L, Chen L, Yuan H-M, Hu H (2024) Efficacy of a single low dose of esketamine after childbirth for mothers with symptoms of prenatal depression: randomised clinical trial. BMJ 385:e078218 38808490 10.1136/bmj-2023-078218 PMC11957566

[47] Johnston JN, Kadriu B, Kraus C, Henter ID, Zarate CA Jr (2024) Ketamine in neuropsychiatric disorders: an update. Neuropsychopharmacology 49:23–40 37340091 10.1038/s41386-023-01632-1 PMC10700638

[48] Jones JL, Mateus CF, Malcolm RJ, Brady KT, Back SE (2018) Efficacy of ketamine in the treatment of substance use disorders: a systematic review. Front Psychiatry 9:372390 10.3389/fpsyt.2018.00277 PMC6094990 30140240

[49] Keeler JL, Treasure J, Juruena MF, Kan C, Himmerich H (2021) Ketamine as a treatment for anorexia nervosa: a narrative review. Nutrients 13:4158 34836413 10.3390/nu13114158 PMC8625822

[50] Jelen LA, McShane R, Young AH (2024) Guidelines for ketamine use in clinical psychiatry practice. BJPsych Open 10:e107 38725375 10.1192/bjo.2024.62 PMC11094435

[51] Lapidus KA, Levitch CF, Perez AM, Brallier JW, Parides MK, Soleimani L, Feder A, Iosifescu DV, Charney DS, Murrough JW (2014) A randomized controlled trial of intranasal ketamine in major depressive disorder. Biol Psychiatry 76:970–976 24821196 10.1016/j.biopsych.2014.03.026 PMC4185009

[52] Kaube H, Herzog J, Kaufer T, Dichgans M, Diener H (2000) Aura in some patients with familial hemiplegic migraine can be stopped by intranasal ketamine. Neurology 55:139–141 10891926 10.1212/wnl.55.1.139

[53] Huge V, Lauchart M, Magerl W, Schelling G, Beyer A, Thieme D, Azad SC (2010) Effects of low-dose intranasal (s)-ketamine in patients with neuropathic pain. Eur J Pain 14:387–394 19733106 10.1016/j.ejpain.2009.08.002

[54] Carr DB, Goudas LC, Denman WT, Brookoff D, Staats PS, Brennen L, Green G, Albin R, Hamilton D, Rogers MC (2004) Safety and efficacy of intranasal ketamine for the treatment of breakthrough pain in patients with chronic pain: a randomized, double-blind, placebo-controlled, crossover study. Pain 108:17–27 15109503 10.1016/j.pain.2003.07.001

[55] Andrade C (2017) Ketamine for depression, 1: clinical summary of issues related to efficacy, adverse effects, and mechanism of action. J Clin Psychiatry 78:10108 10.4088/JCP.17f11567 28448702

[56] d’Andrea G, Pettorruso M, Di Lorenzo G, Rhee TG, Chiappini S, Carullo R, Barlati S, Zanardi R, Rosso G, Di Nicola M (2024) The rapid antidepressant effectiveness of repeated dose of intravenous ketamine and intranasal esketamine: a post-hoc analysis of pooled real-world data. J Affect Disord 348:314–322 38145840 10.1016/j.jad.2023.12.038

[57] Correia-Melo FS, Leal GC, Vieira F, Jesus-Nunes AP, Mello RP, Magnavita G, Caliman-Fontes AT, Echegaray MV, Bandeira ID, Silva SS (2020) Efficacy and safety of adjunctive therapy using esketamine or racemic ketamine for adult treatment-resistant depression: a randomized, double-blind, non-inferiority study. J Affect Disord 264:527–534 31786030 10.1016/j.jad.2019.11.086

[58] Nikayin S, Rhee TG, Cunningham ME (2022) Evaluation of the trajectory of depression severity with ketamine and esketamine in a clinical setting. JAMA Psychiatry 79:736–738 35544190 10.1001/jamapsychiatry.2022.1074 PMC9096687

[59] Singh B, Kung S, Pazdernik V, Schak KM, Geske J, Schulte PJ, Frye MA, Vande Voort JL (2023) Comparative effectiveness of intravenous ketamine and intranasal esketamine in clinical practice among patients with treatment-refractory depression: an observational study. J Clin Psychiatry 84:22m14548 36724113 10.4088/JCP.22m14548

[60] Singh B, Kung S, Vande Voort JL (2024) Intravenous (iv) ketamine versus intranasal esketamine for depression — advantage iv ketamine? J Affect Disord 356:564–567 38657763 10.1016/j.jad.2024.04.088

[61] Calder CN, Kwan ATH, Teopiz KM, Wong S, Rosenblat JD, Mansur RB, Rhee TG, Ho R, Cao B, McIntyre RS (2024) Number needed to treat (nnt) for ketamine and esketamine in adults with treatment-resistant depression: a systematic review and meta-analysis. J Affect Disord 356:753–762 38636712 10.1016/j.jad.2024.04.039

[62] Singh JB, Fedgchin M, Daly E, Xi L, Melman C, De Bruecker G, Tadic A, Sienaert P, Wiegand F, Manji H (2016) Intravenous esketamine in adult treatment-resistant depression: a double-blind, double-randomization, placebo-controlled study. Biol Psychiatry 80:424–431 26707087 10.1016/j.biopsych.2015.10.018

[63] Su T-P, Chen M-H, Li C-T, Lin W-C, Hong C-J, Gueorguieva R, Tu P-C, Bai Y-M, Cheng C-M, Krystal JH (2017) Dose-related effects of adjunctive ketamine in Taiwanese patients with treatment-resistant depression. Neuropsychopharmacology 42:2482–2492 28492279 10.1038/npp.2017.94 PMC5686503

[64] Fava M, Freeman MP, Flynn M, Judge H, Hoeppner BB, Cusin C, Ionescu DF, Mathew SJ, Chang LC, Iosifescu DV (2020) Double-blind, placebo-controlled, dose-ranging trial of intravenous ketamine as adjunctive therapy in treatment-resistant depression (trd). Mol Psychiatry 25:1592–1603 30283029 10.1038/s41380-018-0256-5 PMC6447473

[65] Moaddel R, Abdrakhmanova G, Kozak J, Jozwiak K, Toll L, Jimenez L, Rosenberg A, Tran T, Xiao Y, Zarate CA (2013) Sub-anesthetic concentrations of (r, s)-ketamine metabolites inhibit acetylcholine-evoked currents in α7 nicotinic acetylcholine receptors. Eur J Pharmacol 698:228–234 23183107 10.1016/j.ejphar.2012.11.023 PMC3534778

[66] Daly EJ, Singh JB, Fedgchin M, Cooper K, Lim P, Shelton RC, Thase ME, Winokur A, Van Nueten L, Manji H (2018) Efficacy and safety of intranasal esketamine adjunctive to oral antidepressant therapy in treatment-resistant depression: a randomized clinical trial. JAMA Psychiatry 75:139–148 29282469 10.1001/jamapsychiatry.2017.3739 PMC5838571

[67] Canuso CM, Singh JB, Fedgchin M, Alphs L, Lane R, Lim P, Pinter C, Hough D, Sanacora G, Manji H (2019) Efficacy and safety of intranasal esketamine for the rapid reduction of symptoms of depression and suicidality in patients at imminent risk for suicide: results of a double-blind, randomized, placebo-controlled study. FOCUS 17:55–65 32015715 10.1176/appi.focus.17105 PMC6996073

[68] Fedgchin M, Trivedi M, Daly EJ, Melkote R, Lane R, Lim P, Vitagliano D, Blier P, Fava M, Liebowitz M, Ravindran A, Gaillard R, Ameele HVD, Preskorn S, Manji H, Hough D, Drevets WC, Singh JB (2019) Efficacy and safety of fixed-dose esketamine nasal spray combined with a new oral antidepressant in treatment-resistant depression: results of a randomized, double-blind, active-controlled study (transform-1). Int J Neuropsychopharmacol 22:616–630 31290965 10.1093/ijnp/pyz039 PMC6822141

[69] Popova V, Daly EJ, Trivedi M, Cooper K, Lane R, Lim P, Mazzucco C, Hough D, Thase ME, Shelton RC (2019) Efficacy and safety of flexibly dosed esketamine nasal spray combined with a newly initiated oral antidepressant in treatment-resistant depression: a randomized double-blind active-controlled study. Am J Psychiatry 176:428–438 31109201 10.1176/appi.ajp.2019.19020172

[70] Mahase E (2019) Esketamine is approved in Europe for treating resistant major depressive disorder. BMJ 367:17069 10.1136/bmj.l7069 31862692

[71] Fu D-J, Ionescu DF, Li X, Lane R, Lim P, Sanacora G, Hough D, Manji H, Drevets WC, Canuso CM (2020) Esketamine nasal spray for rapid reduction of major depressive disorder symptoms in patients who have active suicidal ideation with intent: Double-blind, randomized study (aspire i). J Clin Psychiatry 81:6605 10.4088/JCP.19m13191 32412700

[72] Ionescu DF, Fu D-J, Qiu X, Lane R, Lim R, Kasper S, Hough D, Drevets WC, Manji H, Canuso CM (2021) Esketamine nasal spray for rapid reduction of depressive symptoms in patients with major depressive disorder who have active suicide ideation with intent: results of a phase 3, double-blind, randomized study (aspire ii). Int J Neuropsychopharmacol 24:22–31 32861217 10.1093/ijnp/pyaa068 PMC7816667

[73] Reif A, Bitter I, Buyze J, Cebulla K, Frey R, Fu D-J, Ito T, Kambarov Y, Llorca P-M, Oliveira-Maia AJ, Messer T, Mulhern-Haughey S, Rive B, von Holt C, Young AH, Godinov Y, Investigators ESCAPE-TRD (2023) Esketamine nasal spray versus quetiapine for treatment-resistant depression. N Engl J Med 389:1298–1309 37792613 10.1056/NEJMoa2304145

[74] Nijs M, Wajs E, Aluisio L, Turkoz I, Daly E, Janik A, Borentain S, Singh JB, DiBernardo A, Wiegand F (2020) Managing esketamine treatment frequency toward successful outcomes: analysis of phase 3 data. Int J Neuropsychopharmacol 23:426–433 32270176 10.1093/ijnp/pyaa027 PMC7387766

[75] Zaki N, Chen LN, Lane R, Doherty T, Drevets WC, Morrison RL, Sanacora G, Wilkinson ST, Popova V, Fu D-J (2023) Long-term safety and maintenance of response with esketamine nasal spray in participants with treatment-resistant depression: interim results of the sustain-3 study. Neuropsychopharmacology 48:1225–1233 37173512 10.1038/s41386-023-01577-5 PMC10267177

[76] Castro M, Wilkinson ST, Al Jurdi RK, Petrillo MP, Zaki N, Borentain S, Fu D-J, Turkoz I, Sun L, Brown B, Cabrera P (2023) Efficacy and safety of esketamine nasal spray in patients with treatment-resistant depression who completed a second induction period: analysis of the ongoing sustain-3 study. CNS Drugs 37:715–723 37558912 10.1007/s40263-023-01026-3 PMC10439056

[77] Medeiros GC, Gould TD, Prueitt WL, Nanavati J, Grunebaum MF, Farber NB, Singh B, Selvaraj S, Machado-Vieira R, Achtyes ED (2022) Blood-based biomarkers of antidepressant response to ketamine and esketamine: a systematic review and meta-analysis. Mol Psychiatry 27:3658–3669 35760879 10.1038/s41380-022-01652-1 PMC9933928

[78] Liu P, Choi Y-K, Qi RZ (2014) Nme7 is a functional component of the γ-tubulin ring complex. Mol Biol Cell 25:2017–2025 24807905 10.1091/mbc.E13-06-0339 PMC4072575

[79] Li QS, Wajs E, Ochs-Ross R, Singh J, Drevets WC (2020) Genome-wide association study and polygenic risk score analysis of esketamine treatment response. Sci Rep 10:12649 32724131 10.1038/s41598-020-69291-6 PMC7387452

[80] Rotroff D, Corum D, Motsinger-Reif A, Fiehn O, Bottrel N, Drevets W, Singh J, Salvadore G, Kaddurah-Daouk R (2016) Metabolomic signatures of drug response phenotypes for ketamine and esketamine in subjects with refractory major depressive disorder: new mechanistic insights for rapid acting antidepressants. Transl Psychiatry 6:e894–e894 27648916 10.1038/tp.2016.145 PMC5048196

[81] Kumar R, Nuñez NA, Joshi N, Joseph B, Verde A, Seshadri A, Cuellar Barboza AB, Prokop LJ, Medeiros GC, Singh B (2024) Metabolomic biomarkers for (r, s)-ketamine and (s)‐ketamine in treatment‐resistant depression and healthy controls: A systematic review. Bipolar Disord Feb 7 [online ahead of print] 10.1111/bdi.13412 38326104

[82] Liu P, Li P, Li Q, Yan H, Shi X, Liu C, Zhang Y, Peng S (2021) Effect of pretreatment of s-ketamine on postoperative depression for breast cancer patients. J Invest Surg 34:883–888 31948296 10.1080/08941939.2019.1710626

[83] Wang J, Wang Y, Xu X, Peng S, Xu F, Liu P (2020) Use of various doses of s-ketamine in treatment of depression and pain in cervical carcinoma patients with mild/moderate depression after laparoscopic total hysterectomy. Med Sci Monit 26:e922028–e922021 32565534 10.12659/MSM.922028 PMC7331479

[84] Luo T, Deng Z, Ren Q, Mu F, Zhang Y, Wang H (2024) Effects of esketamine on postoperative negative emotions and early cognitive disorders in patients undergoing non-cardiac thoracic surgery: a randomized controlled trial. J Clin Anesth 95:111447 38522144 10.1016/j.jclinane.2024.111447

[85] Liu T, Zhang X, Li A, Liu T, Yang X, Zhang H, Lei Y, Yang Q, Dong H (2023) Effects of intra-operative administration of subanesthetic s-ketamine on emergence from sevoflurane anesthesia: a randomized double-blind placebo-controlled study. BMC Anesthesiol 23:221 37353750 10.1186/s12871-023-02170-5 PMC10288804

[86] Gärtner M, de Rover M, Václavů L, Scheidegger M, van Osch MJ, Grimm S (2022) Increase in thalamic cerebral blood flow is associated with antidepressant effects of ketamine in major depressive disorder. World J Biol Psychiatry 23:643–652 34985394 10.1080/15622975.2021.2020900

[87] Zacharias N, Musso F, Müller F, Lammers F, Saleh A, London M, de Boer P, Winterer G (2020) Ketamine effects on default mode network activity and vigilance: a randomized, placebo-controlled crossover simultaneous fmri/eeg study. Hum Brain Mapp 41:107–119 31532029 10.1002/hbm.24791 PMC7268043

[88] Höflich A, Kraus C, Pfeiffer RM, Seiger R, Rujescu D, Zarate CA Jr, Kasper S, Winkler D, Lanzenberger R (2021) Translating the immediate effects of s-ketamine using hippocampal subfield analysis in healthy subjects-results of a randomized controlled trial. Transl Psychiatry 11:200 33795646 10.1038/s41398-021-01318-6 PMC8016970

[89] Danyeli LV, Sen ZD, Colic L, Kurzweil L, Gensberger-Reigl S, Macharadze T, Götting F, Refisch A, Liebe T, Chand T (2023) Association of the delayed changes in glutamate levels and functional connectivity with the immediate network effects of s-ketamine. Transl Psychiatry 13:60 36797238 10.1038/s41398-023-02346-0 PMC9935558

[90] Danyeli LV, Sen ZD, Colic L, Opel N, Refisch A, Blekic N, Macharadze T, Kretzschmar M, Munk MJ, Gaser C (2024) Cortical thickness of the posterior cingulate cortex is associated with the ketamine-induced altered sense of self: an ultra-high field mri study. J Psychiatr Res 172:136–143 38382237 10.1016/j.jpsychires.2024.02.019

[91] Bojesen KB, Andersen KA, Rasmussen SN, Rostrup E (2018) Glutamate levels and resting cerebral blood flow in anterior cingulate cortex are associated at rest and immediately following infusion of s-ketamine in healthy volunteers. Front Psychiatry 9:306440 10.3389/fpsyt.2018.00022 PMC5808203 29467681

[92] Gärtner M, Aust S, Bajbouj M, Fan Y, Wingenfeld K, Otte C, Heuser-Collier I, Böker H, Hättenschwiler J, Seifritz E (2019) Functional connectivity between prefrontal cortex and subgenual cingulate predicts antidepressant effects of ketamine. Eur Neuropsychopharmacol 29:501–508 30819549 10.1016/j.euroneuro.2019.02.008

[93] Herrera-Melendez A, Stippl A, Aust S, Scheidegger M, Seifritz E, Heuser-Collier I, Otte C, Bajbouj M, Grimm S, Gärtner M (2021) Gray matter volume of rostral anterior cingulate cortex predicts rapid antidepressant response to ketamine. Eur Neuropsychopharmacol 43:63–70 33309459 10.1016/j.euroneuro.2020.11.017

[94] Weigand A, Gärtner M, Scheidegger M, Wyss PO, Henning A, Seifritz E, Stippl A, Herrera-Melendez A, Bajbouj M, Aust S (2022) Predicting antidepressant effects of ketamine: the role of the pregenual anterior cingulate cortex as a multimodal neuroimaging biomarker. Int J Neuropsychopharmacol 25:1003–1013 35948274 10.1093/ijnp/pyac049 PMC9743970

[95] Bera K, Kamajaya A, Shivange AV, Muthusamy AK, Nichols AL, Borden PM, Grant S, Jeon J, Lin E, Bishara I (2019) Biosensors show the pharmacokinetics of s-ketamine in the endoplasmic reticulum. Front Cell Neurosci 13:499 31798415 10.3389/fncel.2019.00499 PMC6874132

[96] Altê GA, Rodrigues ALS (2023) Exploring the molecular targets for the antidepressant and antisuicidal effects of ketamine enantiomers by using network pharmacology and molecular docking. Pharmaceuticals (Basel) 16:1013 37513925 10.3390/ph16071013 PMC10383558

[97] Vankawala J, Naples G, Avila-Quintero VJ, Ramirez KL, Flores JM, Bloch MH, Dwyer JB (2021) Meta-analysis: hemodynamic responses to sub-anesthetic doses of ketamine in patients with psychiatric disorders. Front Psychiatry 12:549080 33841195 10.3389/fpsyt.2021.549080 PMC8024485

[98] Khalili-Mahani N, Martini C, Olofsen E, Dahan A, Niesters M (2015) Effect of subanaesthetic ketamine on plasma and saliva cortisol secretion. Br J Anaesth 115:68–75 25982133 10.1093/bja/aev135

[99] Toki H, Yamaguchi J-i, Mizuno-Yasuhira A, Endo H (2023) Chiral lc-ms/ms method for the simultaneous determination of (r, s)-ketamine,(r, s)-norketamine, and (2r, 6r; 2s, 6s)-hydroxynorketamine in mouse plasma and brain. J Pharm Biomed Anal 224:115168 36473323 10.1016/j.jpba.2022.115168

[100] Hasan M, Modess C, Roustom T, Dokter A, Grube M, Link A, Rey H, Adler S, Meissner K, Siegmund W (2021) Chiral pharmacokinetics and metabolite profile of prolonged-release ketamine tablets in healthy human subjects. Anesthesiology 135:326–339 34019627 10.1097/ALN.0000000000003829

[101] Gilbert JR, Yarrington JS, Wills KE, Nugent AC, Zarate CA Jr (2018) Glutamatergic signaling drives ketamine-mediated response in depression: evidence from dynamic causal modeling. Int J Neuropsychopharmacol 21:740–747 29668918 10.1093/ijnp/pyy041 PMC6070027

[102] Fagerholm ED, Leech R, Williams S, Zarate CA Jr, Moran RJ, Gilbert JR (2021) Fine-tuning neural excitation/inhibition for tailored ketamine use in treatment-resistant depression. Transl Psychiatry 11:335 34052834 10.1038/s41398-021-01442-3 PMC8164631

[103] Medeiros GC, Matheson M, Demo I, Reid MJ, Matheson S, Twose C, Smith GS, Gould TD, Zarate CA, Barrett FS (2023) Brain-based correlates of antidepressant response to ketamine: a comprehensive systematic review of neuroimaging studies. Lancet Psychiatry 10:790–800 37625426 10.1016/S2215-0366(23)00183-9 PMC11534374

[104] Nunes RR, Akamine FM, Meireles BR, de Moraes Nobre DG, Nascimento JCR (2023) Influence of s-ketamine, at low doses, on the electroencephalogram-bis suppression rate: a randomized clinical trial. J Surg Anesth Res 163:2–5

[105] Frolich J, Van Horn JD (2014) Reviewing the ketamine model for schizophrenia. J Psychopharmacol 28:287–302 24257811 10.1177/0269881113512909 PMC4133098

[106] Liu L-L, Hu J-H, Pan J-J, Liu H, Ji F-H, Peng K (2023) An intraoperative sub-anesthetic dose of esketamine on postoperative depressive symptoms in perimenopausal women with breast cancer undergoing modified radical mastectomy: protocol for a randomized, triple-blinded, controlled trial. Int J Gen Med 16:3373–3381 37576915 10.2147/IJGM.S421265 PMC10422984

[107] Frye MA, Blier P, Tye SJ (2015) Concomitant benzodiazepine use attenuates ketamine response: implications for large scale study design and clinical development. J Clin Psychopharmacol 35:334–336 25928701 10.1097/JCP.0000000000000316

[108] Albott CS, Shiroma PR, Cullen KR, Johns B, Thuras P, Wels J, Lim KO (2017) The antidepressant effect of repeat dose intravenous ketamine is delayed by concurrent benzodiazepine use. J Clin Psychiatry 78:1863 10.4088/JCP.16l11277 28394513

[109] Grunebaum MF, Galfalvy HC, Choo T-H, Keilp JG, Moitra VK, Parris MS, Marver JE, Burke AK, Milak MS, Sublette ME (2018) Ketamine for rapid reduction of suicidal thoughts in major depression: a midazolam-controlled randomized clinical trial. Am J Psychiatry 175:327–335 29202655 10.1176/appi.ajp.2017.17060647 PMC5880701

[110] Nikayin S, Murphy E, Krystal JH, Wilkinson ST (2022) Long-term safety of ketamine and esketamine in treatment of depression. Expert Opin Drug Saf 21:777–787 35416105 10.1080/14740338.2022.2066651

[111] Souza-Marques B, Santos-Lima C, Araujo-de-Freitas L, Vieira F, Jesus-Nunes AP, Quarantini LC, Sampaio AS (2021) Neurocognitive effects of ketamine and esketamine for treatment-resistant major depressive disorder: a systematic review. Harv Rev Psychiatry 29:340–350 34366408 10.1097/HRP.0000000000000312

[112] Lan X, Wang C, Zhang F, Liu H, Li W, Ye Y, Hu Z, Mai S, Ning Y, Zhou Y (2023) Short-term cognitive effects of repeated-dose esketamine in adolescents with major depressive disorder and suicidal ideation: a randomized controlled trial. Child Adolesc Psychiatry Ment Health 17:108 37710297 10.1186/s13034-023-00647-2 PMC10503003

[113] Pepe M, Bartolucci G, Marcelli I, Simonetti A, Camardese G, Di Nicola M, Sani G (2023) Reduction in cognitive symptoms following intranasal esketamine administration in patients with chronic treatment-resistant depression: a 12-week case series. J Psychiatr Pract 29:325–332 37449831 10.1097/PRA.0000000000000723

[114] van de Loo AJ, Bervoets AC, Mooren L, Bouwmeester NH, Garssen J, Zuiker R, van Amerongen G, van Gerven J, Singh J, der Ark PV (2017) The effects of intranasal esketamine (84 mg) and oral mirtazapine (30 mg) on on-road driving performance: a double-blind, placebo-controlled study. Psychopharmacology 234:3175–3183 28755104 10.1007/s00213-017-4706-6 PMC5660834

[115] Solomon TM, Hajduk M, Majernik M, Jemison J, Deschamps A, Scoggins J, Kolar A, Pinheiro MA, Dubec P, Skala O (2023) Evaluating passive physiological data collection during spravato treatment. Front Digit Health 5:1281529 38094111 10.3389/fdgth.2023.1281529 PMC10716422

[116] Baudot J, Soeiro T, Tambon M, Navarro N, Veyrac G, Mezaache S, Micallef J (2022) Safety concerns on the abuse potential of esketamine: multidimensional analysis of a new anti-depressive drug on the market. Fundam Clin Pharmacol 36:572–581 34907579 10.1111/fcp.12745

[117] Halaris A, Cook J (2023) The glutamatergic system in treatment-resistant depression and comparative effectiveness of ketamine and esketamien: role of inflammation? In: Kim YK (ed) Neuroinflammation, gut-brain axis and immunity in neuropsychiatric disorders advances in experimental medicine and biology. Springer, Singapore, pp 487–512 10.1007/978-981-19-7376-5_21 36949323

